# Understanding pine wilt disease: roles of the pine endophytic bacteria and of the bacteria carried by the disease‐causing pinewood nematode

**DOI:** 10.1002/mbo3.415

**Published:** 2016-10-26

**Authors:** Diogo N. Proença, Gregor Grass, Paula V. Morais

**Affiliations:** ^1^ CEMUCUniversity of CoimbraCoimbraPortugal; ^2^ Department of Biology and CESAMUniversity of AveiroAveiroPortugal; ^3^ Bundeswehr Institute of MicrobiologyMunichGermany; ^4^ Department of Life SciencesUniversity of CoimbraCoimbraPortugal

**Keywords:** bacteria, biocontrol, *Bursaphelenchus xylophilus*, endophytes, nematodes, pine wilt disease

## Abstract

Pine wilt disease (PWD) is one of the most destructive diseases in trees of the genus *Pinus* and is responsible for environmental and economic losses around the world. The only known causal agent of the disease is the pinewood nematode (PWN) *Bursaphelenchus xylophilus*. Despite that, bacteria belonging to several different genera have been found associated with PWN and their roles in the development of PWD have been suggested. Molecular methodologies and the new era of genomics have revealed different perspectives to the problem, recognizing the manifold interactions between different organisms involved in the disease. Here, we reviewed the possible roles of nematode‐carried bacteria in PWD, what could be the definition of this group of microorganisms and questioned their origin as possible endophytes, discussing their relation within the endophytic community of pine trees. The diversity of the nematode‐carried bacteria and the diversity of pine tree endophytes, reported until now, is revised in detail in this review. What could signify a synergetic effect with PWN harming the plant, or what could equip bacteria with functions to control the presence of nematodes inside the tree, is outlined as two possible roles of the microbial community in the etiology of this disease. An emphasis is put on the potential revealed by the genomic data of isolated organisms in their potential activities as effective tools in PWD management.

## 1 Introduction

Pine wilt disease (PWD) is one of the most destructive diseases of trees of the genus *Pinus* and is responsible for environmental and economic losses around the world accruing to tens of million dollars (Tóth, 2011). The etiology of the disease has not been well understood, but generally browning of plant tissues is caused by oxidation of phenols which occurs as a result of cellular disorganization (Pirttilä, Podolich, Koskimäki, Hohtola, & Hohtola, 2008; Whitaker & Lee, 1995). The accumulation of terpenes in xylem tissue resulted in cavitation, which interrupts the water flux in the pine trees (Kuroda, 1991). The development of PWD coincides with increasing number of brown pine needles. The only known causal agent of the disease is the pinewood nematode (PWN) *Bursaphelenchus xylophilus* (Nickle, Golden, Mamiya, & Wergin, 1981).

**Figure 1:**
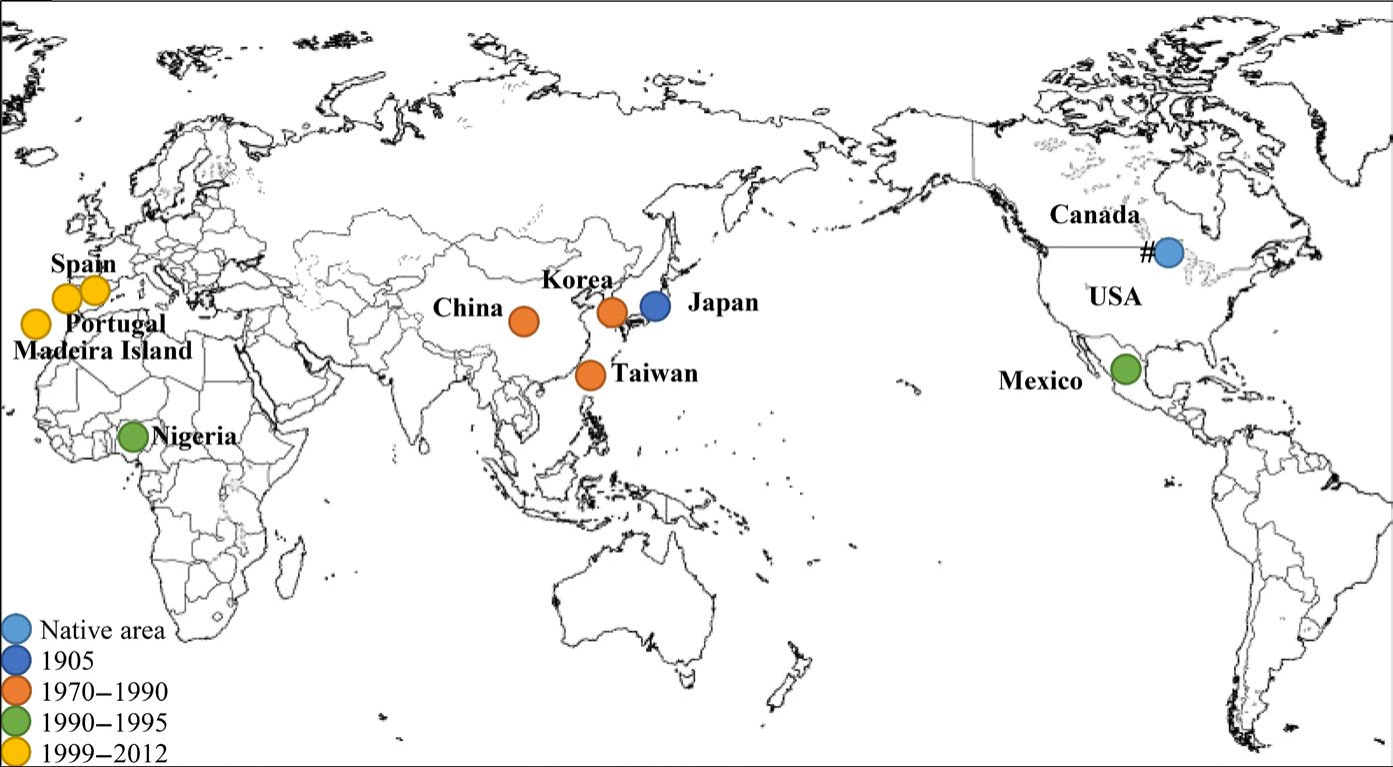
Temporal and spatial spreading of pine wilt disease around the world. The PWN is native to North America (light blue) and was spread into Japan (blue); China, Korea, and Taiwan (orange); Nigeria and Mexico (green); and Portugal (continental and Madeira island) and Spain (yellow). ^#^Occurrence of the disease in forests in North America is mostly limited to nonnative tree species

Native to North America (USA and Canada), PWN was introduced and first reported in Japan at the beginning of the 20th century and has been spread into China, Korea, Taiwan in the late 1970–1980s, and thereafter to Europe (Portugal and Spain) (EPPO/OEPP 2009; Evans, McNamara, Braasch, Chadoeuf, & Magnusson, 1996; Fonseca et al., 2012; Mamiya, 1988; Mota et al., 1999; Nickle et al., 1981; Robertson et al., 2011; Sutherland & Webster, 1993; Zhao, Futai, Sutherland, & Takeuchi, 2008) (Figure 1). Moreover, PWN was also reported in Nigeria and Mexico (Dwinnel, 1993; Khan & Gbadegesin, 1991).

*Bursaphelenchus xylophilus* was initially classified as *Aphelenchoides xylophilus* by Steiner & Buhrer, 1934 from blue‐stained logs of *P. palustris* in Louisiana, USA, but was later reclassified by Nickle et al. (1981) on the basis of morphological characters. The genus *Bursaphelenchus* is a member of the phylum Nematoda, reclassified by Hunt (2008) in the family Aphelenchoididae (Rhabditida: Tylenchomorpha) which includes species with various feeding habitats (Hunt, 2008).

The transmission of PWN between host trees is mediated by insect vectors. The PWD vectors, pine sawyer beetles of the genus *Monochamus* comprise different species, belonging to the family *Cerambycidae* and constitutes the main element in the epidemiology of PWD. In Japan, the major vector is *Monochamus alternatus*, in North America *M. carolinensis*, and in Portugal, *M. galloprovincialis* is the only vector (Evans et al., 1996; Linit, 1988; Mamiya & Enda, 1972; Sousa et al., 2001). The role of the insect in the disease was reviewed by Zhao, Mota, Vieira, Butcher, and Sun (2014).

The PWN and *Monochamus* spp. were initially listed as European and Mediterranean Plant Protection Organization (EPPO) A1 quarantine pests (EPPO/OEPP 2009). Recently, PWN was reclassified as A2 in Portugal (http://www.eppo.int/QUARANTINE/quarantine.htm). For each country, A2 pests list includes organisms already present in contrast to A1 species which are present in some of the countries belonging to EPPO. The purpose of EPPO A1/A2 lists were to regulate phytosanitary concerns of quarantine pests and to raise awareness for plant species in EPPO member countries posing a threat to plant health, biodiversity, and the environment.

The bacterial community in pine trees affected by PWD have been studied (Paiva et al., 2013; Proença et al., 2010, 2011; Vicente et al., 2012; Vicente, Ikuyo, Mota, & Hasegawa, 2013; Vicente et al., 2013; Wu et al., 2013) as early as when the presence of endophytes in plant tissues had been recognized as positively relevant for the trees (Rosenblueth & Martínez‐Romero, 2006; Ryan, Germaine, Franks, Ryan, & Dowling, 2008). However, the observation of increasing numbers of bacteria in the tissues of trees affected by PWD led to more recent research suggesting that bacteria may be participants in PWD (Guo, Guo, Zhao, Xu, & Li, 2007; Han, Hong, & Zhao, 2003; Higgins, Harmey, & Jones, 1999; Oku, Shiraishi, Ouchi, Kurozumi, & Ohta, 1980; Proença et al., 2010; Zhao et al., 2009). In 2005, a mutualistic role of PWN and the bacteria it carries was proposed (Zhao & Lin, 2005). This relationship, however, is obscured because bacteria from the same species have also been founded as intrinsic parts of the endophytic microbial community of healthy *Pinus* spp. trees (Bal, Anand, Berge, & Chanway, 2012; Carrell & Frank, 2014; Izumi, Anderson, Killham, & Moore, 2008; Pirttilä, Laukkanen, Pospiech, Myllylä, & Hohtola, 2000; Pirttilä et al., 2008; Proença et al., 2011; Shishido, Loeb, & Chanway, 1995; Strzelczyk & Li, 2000). On the other hand, bacterial communities from the insect vectors were assessed, but a causality between PWN and insect vectors' bacterial communities was not firmly established (Vicente, Ikuyo, et al., 2013; Vicente et al., 2013). Table 1 gives a brief summary, by country, of the species of *Bursaphelenchus* for which data related with the nematode‐carried bacteria and the insect vector and its microbiome were found.

**Table 1:** Known data of associations of the wild nematodes *Bursaphelenchus*, bacteria, and insect vectors of the genus *Monochamus*

Isolation country	Nematode	Bacteria carried by wild nematode	*Monochamus* spp.	*Monochamus* bacterial diversity
Japan	*B. xylophilus*	Kawazu, Zhang, & Kanzaki (1996)	*M. alternatus*	Alves et al. (2016)
		Oku et al. (1980)		
China	*B. xylophilus*	Han et al. (2003)	*M. alternatus*	Wang, Xu, Jiang, Zhang, and Yang (2004)
		Zhao et al. (2003)		
		Zhao and Lin (2005)		
		Tian et al. (2010)		
		Wu et al. (2013)		
		Cheng et al. (2013)		
	*B. mucronatus*	Xiang et al. (2015)		No data
Korea	*B. xylophilus*	Kwon et al. (2010)	*M. alternatus*	
Portugal	*B. xylophilus*	Proença et al. (2010)	*M. galloprovincialis*	Alves et al. (2016)
		Vicente et al. (2011)		Vicente, Ikuyo, et al. (2013); Vicente et al. (2013)
	*B. mucronatus*	ND		
USA	*B. xylophilus*	Proença, Fonseca, et al. (2014)	*M. alternatus*	No data
			*M. titillator*	
			*M. carolinensis*	
			*M. scutellatus*	
			*M. obtusus*	
Russia	*B. mucronatus*	Arbuzova et al. (2016)	*M. urussovi*	No data

Here, we reviewed the possible roles of nematode‐carried bacteria in PWD, what could be the definition of this group of microorganisms, and discuss their origin and relation with the endophytic community of pine trees. Finally, we assessed the potential signified by the genomic data of isolated PWN‐associated bacteria for their potential activities as effective tools in PWD management.

## 2 PWD Development

**Figure 2:**
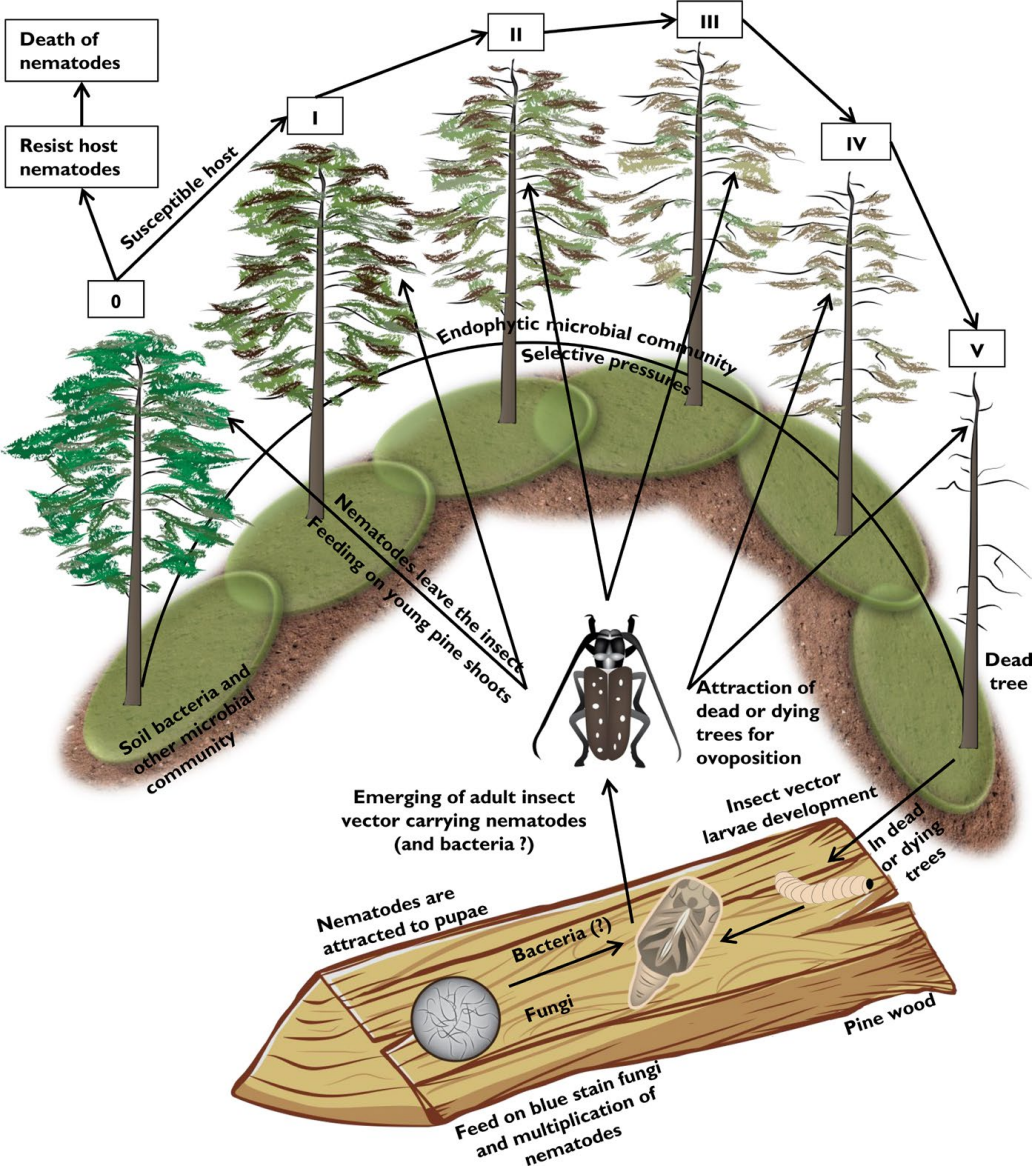
Schematic depiction of relationships between *B. xylophilus and Monochamus spp*. life cycles and pine wilt disease (PWD) development. The insect vector is attracted by healthy trees and releases nematodes during maturation feeding on young pine shoots. The pine trees could be resistant (death of nematodes) or susceptible (development of PWD symptoms, culminating in the death of pine trees). *Monochamus* spp. are also attracted by dead or dying pine trees for ovoposition. Later, during the larvae development, nematodes are attracted to pupae and (?) indicates that the bacteria maybe carried by nematodes into *Monochamus* spp. but this remains untested. The cycle restarts by emerging of adult *Monochamus* spp. carrying nematodes (and probably bacteria). PWD symptom classes: 0—tree without symptoms; I—‹10% brown leaves; II—10‐50% brown leaves; III—50‐80% brown leaves; IV—› 80% brown leaves; V—dead tree without leaves

Diverse species of the genus *Pinus* are the main host for PWN (Evans et al., 1996). However, some other tree genera such as *Abies*,* Chamaecyparis*,* Cedrus*,* Larix*,* Picea*, or *Pseudotsuga* have also been reported as permissive hosts for the complete life cycle of PWN. The life cycle of PWN and PWD development is summarized in Figure 2. *Monochamus* spp. carry *B. xylophilus* inside their tracheal system and on their body surfaces. Attracted by healthy trees, the insect vector releases nematodes during maturation feeding on young pine shoots (primary transmission). Whether disease occurs depends on the *Pinus* spp. tree being resistant or susceptible to the multiplication of the nematodes (Jones, Moens, Mota, Li, & Kikuchi, 2008). Additionally, *Monochamus* spp. are also attracted by dead or dying pine trees for oviposition (secondary transmission). In this instant, insect vector larvae development follows and *B. xylophilus* feed on blue stain fungi (*Grosmanniaclavigera*) which developed as a consequence of insect penetration (Evans et al., 1996). The disease and its three long‐term recognized actors have been reviewed before (Evans et al., 1996; Jones et al., 2008; Nascimento, Hasegawa, Mota, & Vicente, 2015; Zhao et al., 2014).

Before the adult insect vector emerges from its chrysalis, the nematodes are attracted, enter the bug's tracheal system, and restart the cycle (Evans et al., 1996; Linit, 1988; Sousa et al., 2001). According to Zhao, Wei, Kang, and Sun (2007), three terpenes (α‐pinene, β‐pinene, and longifolene) are produced by larval *M. alternatus* and are able to attract nematodes. Moreover, these terpenes were found in the xylem of healthy *Pinus massoniana* in different ratios, compared to the ratio of terpenes produced by larval *M. alternatus,* which might be involved in attraction of the insect vector. These three volatile terpenes are now thought to constitute the basis for a chemoecological relationship between PWN and *M. alternatus* (Zhao et al., 2007).

The global spread of pine wilt disease has been supported by several factors, that is, changing climatic conditions, topographic and biological factors, as well as by human activities, especially infested timber exports (Futai, 2008). In Portugal, the nematode was detected for the first time in 1999 by Mota et al. in the Setúbal area (Mota et al., 1999). Measures were undertaken to control the spread of the disease in this area using a border‐belt areas. However, these efforts were thwarted and PWD spread to the Center‐North of the country. In 2008, PWN was detected in the district of Coimbra (Rodrigues, 2008; Rodrigues, Casquilho, Oliveira, & Bordado, 2009) and in 2012 on Madeira island (Fonseca et al., 2012). The impact of this disease was severe for Portuguese forests, for they are mainly composed of species of pine, eucalyptus, and oak trees. In Portuguese pine forests, the most abundant species with 62.5% of all trees, maritime pine (*P. pinaster*), is a PWD‐susceptible pine species; other minor species in such forests are stone pine (*P. pinea*) and Scots pine (*P. sylvestris*) (Rodrigues, 2008). In 2008, the Portuguese government unified research efforts in order to better understand the disease itself, to control PWN‐spread, all with the ultimate goal to find a solution to this massive ecological and economic challenge (Anon 2008).

When colonizing pine wood, the PWN will be in contact with the tree's endophytic microbial community. If these bacteria interact with the nematode had not yet been explored. Therefore, several studies have been conducted to assess and characterize the diversity of endophytic microbial communities in pine trees. These findings will be central to discuss the relationship of the pathogen with the nematode‐carried bacteria and understand how this interplay can influence the course of PWD.

## 3 Plant–Microbe Interactions

Plants constitute a quite complex microbial ecosystem comprising different tissues or intercellular spaces spanning from the roots up to the shoots and leaves, all of which may be colonized by microbes (reviewed in Reinhold‐Hurek & Hurek, 2011). Comparatively, the density of the microbial community at the rhizosphere is generally higher than the density of the endophytic microbial community that resides within the plants (Compant, Clément, & Sessitsch, 2010; Hallmann, Quadt‐Hallmann, Mahaffee, & Kloepper, 1997). In general, plants feature distinct microbial communities depending on the plant species and these intrinsic microbial communities may strongly interact with the welfare of plants (Ma, Prasad, Rajkumar, & Freitas, 2011; Rosenblueth & Martínez‐Romero, 2006).

Today, we realize that endophytes can be found to reside within roots, stems, and leaves, and bacteria from more than one hundred genera have been identified in a broad range of plants, including woody plants and arable crops (Lodewyckx et al., 2002; Rosenblueth & Martínez‐Romero, 2006; Ryan et al., 2008). Obligate endophytes are usually related to function as nitrogen fixation and the organisms are less diverse then facultative endophytes (Box 1). Bacteria may be “competent” or “opportunistic” endophytes, depending on whether they possess the key genetic machinery required for colonization (Hallmann et al., 1997; Zinniel et al., 2002).

Box 1 **Plant–Microbe Terms**

1 *Endophyte* (“endo” means “inside” and “phyte” means “plant”) was previously defined as ‘an organism inhabiting plant organs, that at some time in its life cycle can colonize internal plant tissue without causing apparent harm to the host' (Petrini, 1991). In addition, endophytic bacteria have been defined as bacteria colonizing the internal plant tissues without causing harm to their host and being able to establish a mutualistic association (Hallmann et al., 1997).

*Obligate endophytes* are strictly dependent on host plants for their survival, no life stages occur outside the plant except for plant‐to‐plant (vertically) or plant‐to‐insect‐to‐plant (horizontally, by insect vectors) transmission to other host plants (Hardoim et al., 2015).

*Facultative endophytes* may complete phases of their life cycles inside or outside plants (Hardoim et al., 2015).

*Competent or opportunistic endophytes* depend on the endophyte possessing the key genetic machinery required for colonization or not (Hallmann et al., 1997; Zinniel et al., 2002).

*Bacterial colonization* of plant tissues can occur in different ways. Usually, some rhizosphere bacteria colonize plants through lateral root outgrowth, and then colonize local root tissues, followed by migration throughout internal plant tissues from stem to leaves (Chi et al., 2005). Another way of bacterial colonization of plant tissues occurs through wounds afflicted to the plant host by external agents, such as nematodes, insects, or other mechanical injuries (Linit, 1988). In contrast, active penetration of endophytic bacteria into plant tissues seems to be accomplished through the production of hydrolytic enzymes (Quadt‐Hallmann et al., 1997).

A variety of phytopathogenic bacteria causing harm and diseases to plant hosts have been reclassified because of advances in taxonomy such as molecular polyphasic, hierarchical approaches for improved phylogeny (Vandamme et al., 1996). Currently, 39 genera are known to include phytopathogenic species (Bull et al., 2010, 2012). Moreover, a study by Mansfield et al. (2012) defined the 10 most relevant plant pathogenic bacteria according to scientific/economic importance. *Pseudomonas syringae* (various pathovars) was the species with the highest score and three species belonging to the genus *Xanthomonas* are present on top of this top list (Mansfield et al., 2012). The class *Gammaproteobacteria* was the most abundant with seven species and these 10 bacterial species should be taken in consideration for researchers working on bacteria–plant interactions.

The ability to cause harm to plants by bacterial pathogens is due to: (i) secretion of wall degradative enzymes, such as cellulases and pectinases (Quadt‐Hallmann, Kloepper, & Benhamou, 1997); (ii) toxin production; (iii) extracellular polysaccharide (EPS) production causing host xylem vessel blockage, for example, by *Xylella fastidiosa* (Roper, Greve, Labavitch, & Kirkpatrick, 2007); (iv) excess of auxin‐causing tumors (Lee et al., 2009); (v) production of effector proteins that block the induced systemic resistance (ISR) (Alfano & Collmer, 2004); vi) production of secretion systems induced by *hrp* (hypersensitive response and pathogenicity) or *avr* (avirulence) genes (Alfano & Collmer, 1996).

However, in forest trees such as pine trees, there are no known diseases associated with the presence of phytopathogenic bacteria. In contrast, pine trees, eucalyptus, and oak trees suffer from various diseases typically caused by fungi, insects, or nematodes (Cooper & Gardener, 2006). The impact of pine tree diseases were described in several papers and partially reviewed by Boyd, Freer‐Smith, Gilligan, and Godfray (2013).

## 4 Endophytic Bacteria in *Pinus* spp

Being one of the most abundant trees in many temperate forests, *Pinus* spp. may constitute vast habitats for bacteria. Thus, in recent years, studies have aimed at investigating the endophytic microbial community of several pine tree species. These studies used methodologies varying from cultivation‐based to biochemical characterization (Strzelczyk & Li, 2000) of the isolates by BIOLOG phenotypic assay and GC‐FAME (Bal et al., 2012; Shishido et al., 1995) to molecular denaturating gradient gel electrophoresis (DGGE) (Izumi et al., 2008) as well as 16S rRNA gene sequencing (Sanger and pyrosequencing) (Bal et al., 2012; Carrell & Frank, 2014; Izumi et al., 2008; Pirttilä et al., 2000; Redford, Bowers, Knight, Linhart, & Fierer, 2010). Therefore, sometimes it is difficult to compare the diversity described in the different studies since these techniques have varying degrees of taxonomic resolution.

The presence of endophytic bacteria has been reported, for example, in *Pinus contorta* (Bal et al., 2012; Shishido et al., 1995), *P. sylvestris* (Izumi et al., 2008; Pirttilä et al., 2000, 2008; Strzelczyk & Li, 2000), and *P. flexilis* (Carrell & Frank, 2014) (Table 2). Not unexpectedly, the diversity of microbial communities of pine trees seems to be different between rhizosphere and root tissue (Izumi et al., 2008).

**Table 2:** Bacterial endophytes identified in their respective *Pinus* host species

Endophyte genera	Endophyte Species^a^	Host pine tree	Plant tissue	Reference
*Acetobacter*	*Acetobacter* sp.	*P. flexilis*	Needle	Carrell and Frank (2014)
*Bacillus*	*Bacillus* sp.	*P. contorta*	Needle	Bal et al. (2012)
			Stem	
			Roots	
	*B. arvi*	*P. sylvestris*	Roots	Izumi et al. (2008)
	*B. licheniformis*	*P. contorta*	Stem	Bal et al. (2012)
	*B. longisporus*		Stem	
	*B. megaterium*		Stem	
	*B. mycoides*		Stem	
	*B. polymyxa*		Roots	Shishido et al. (1995)
	*B. pumilus*		Needle	Bal et al. (2012)
			Stem	
			Roots	
*Brevibacillus*	*Brevibacillus* sp.		Stem	Bal et al. (2012)
*Brevundimonas*	*Brevundimonas vesicularis*		Stem	
*Burkholderia*	*Burkholderia pyrrocinia*		Stem	
*Cellulomonas*	*Cellulomonas biazotea*		Stem	
*Dyadobacter*	*Dyadobacter fermantans*		Stem	
*Gluconacetobacter*	*Gluconacetobacter* sp.	*P. flexilis*	Needle	Carrell and Frank (2014)
*Kocuria*	*Kocuria kristinae*	*P. contorta*	Stem	Bal et al. (2012)
	*K. rosea*		Stem	
*Methylobacterium*	*Methylobacterium extorquens*	*P. sylvestris*	Buds	Pirttilä et al. (2000)
*Paenibacillus*	*Paenibacillus* sp.	*P. contorta*	Needle	Bal et al. (2012)
			Stem	
	*P. gordonae*		Needle	
	*P. pabuli*		Needle	
		*P. sylvestris*	Roots	Izumi et al. (2008)
	*P. peoriae*	*P. contorta*	Stem	Bal et al. (2012)
	*P. polymyxa*		Needle	
			Stem	
	*P. wynnii*	*P. sylvestris*	Roots	Izumi et al. (2008)
*Pseudomonas*	*Pseudomonas sp*.	*P. contorta*	Stem	Bal et al. (2012)
		*P. sylvestris*	Roots	Strzelczyk and Li (2000)
	*P. synxantha*		Buds	Pirttilä et al. (2000)
Genera belonging to *Alphaproteobacteria* (class)		*P. flexilis*	Needle	Carrell and Frank (2014)
Genera belonging to *Acetobacteraceae* (family)			Needle	
Genera belonging to the *Bacteroidetes* (phylum)			Needle	Redford et al. (2010)
Genera belonging to the *Bacteroidetes* (phylum)		*P. ponderosa*	Needle	
		*P. strobiformis*	Needle	
		*P. nigra*	Needle	
		*P. heldreichii*	Needle	

a Bacterial identification techniques: cultivation‐based and biochemical characterization (Strzelczyk & Li, 2000) of the isolates by BIOLOG phenotypic assay and GC‐FAME (Bal et al., 2012; Shishido et al., 1995) to molecular denaturating gradient gel electrophoresis (DGGE) (Izumi et al., 2008) and 16S rRNA gene sequencing (Sanger and pyrosequencing) (Bal et al., 2012; Carrell & Frank, 2014; Izumi et al., 2008; Pirttilä et al., 2000; Redford et al., 2010).

The major bacterial genera in Scots pine, *P. sylvestris,* vary according to different studies, but comprise, for example, *Methylobacterium* (Pirttilä et al., 2000), *Pseudomonas* (Pirttilä et al., 2000; Strzelczyk & Li, 2000), or *Bacillus* and *Paenibacillus* (Izumi et al., 2008). The first study of endophytic bacteria in *Pinus* spp. reported the isolation of a siderophore‐producing *Pseudomonas* from *P. sylvestris*, followed by the study of Pirttilä et al. (2000) in which *Methylobacterium extorquens* and *Pseudomonas synxantha* were found on buds of Scots pines. In the study by Izumi et al. (2008), also on Scots pines, *Paenibacillus pabuli*,* P. wynii*, and *Bacillus arvi* were found. Strains belonging to the genera *Paenibacillus* and *Bacillus* were found as endophytes of Lodgepole pine, *P. contorta* (Bal et al., 2012; Shishido et al., 1995). *Paenibacillus polymyxa* and notably the gram‐negative *Dyadobacter fermentans* were able to reduce acetylene suggesting the ability to fix nitrogen (Bal et al., 2012).

**Figure 3:**
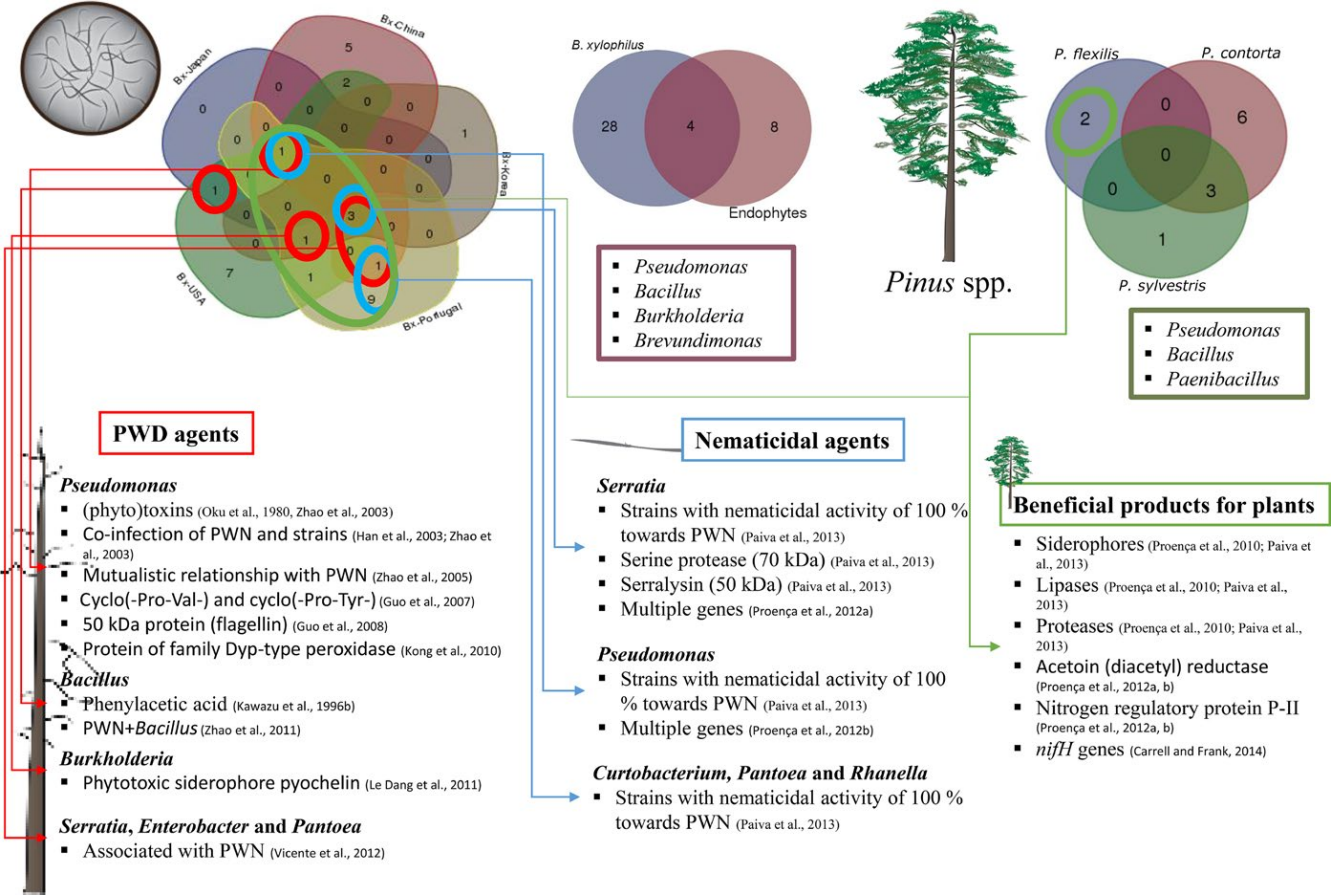
Comprehensive role of bacteria in pine wilt disease (PWD). Venn diagrams showing common bacteria: i) associated with PWN from Portugal, USA, China, Japan, and Korea; ii) associated with PWN and endophytes from *Pinus* spp.; and iii) endophytic from *P. flexilis*,* P. contorta*, and *P. sylvestris*. Diagrams illustrate the three known roles of bacteria related with *B. xylophilus* or pine trees in PWD: i) PWD agents (red circles and red arrows) producing toxins and proteins; ii) nematicidal agents (blue circles and arrows) producing proteases and secretion machinery; and iii) beneficial products for plants (green circles and arrows) such as siderophores, lipases, proteases, acetoin (diacetyl) reductase, and nitrogen fixation genes. Bx ‐ *Bursaphelenchus xylophilus*

More recently, the major foliar endophytic bacterial community in the limber pine, *P. flexilis,* was found to belong to the class *Alphaproteobacteria* (60%) (Carrell & Frank, 2014). The same study also found nitrogen‐fixing bacteria (e.g., genus *Gluconacetobacter*) in the foliar endophytic community by PCR amplification of the *nifH* gene suggesting a role in nitrogen fixation by the endophytic microbial community (Figure 3). Moreover, a comparative study between bacterial communities of leaves from different tree species showed *Bacteroidetes* as the major component of the phylosphere of the *Pinus* spp. (Redford et al., 2010).

In Portugal, the endophytic microbial community of maritime pines, *P. pinaster*, was mainly composed of strains belonging to the family *Gammaproteobacteria*. This microbial community typically harbored *nif* and *nir* genes also suggesting a role in nitrogen fixation and denitrification, respectively. Recently, for the first time, the domain *Archaea* was found as part of an endophytic community of pine trees as determined by DGGE and next‐generation sequencing belonging to the phyla *Euryarchaeota, Thaumarchaeota,* and *Crenarchaeota* (Proença, 2014). Also resulting from this study, two novel endophytic species, *Chitinophaga costaii* and *Mucilaginibacter pineti*, were described (Paiva et al., 2014; Proença, Fonseca, Powers, Abrantes, & Morais, 2014; Proença, Nobre, & Morais, 2014).

Different cultivation‐dependent and ‐independent methodologies were performed in studies described in Table 2 to gain a better understanding of the endophytic microbial diversity. However, it is not clear yet if the overall observed differences in endophytic microbial communities might also be due to differences in soil compositions, other chemical–physical parameters, or different sampling locations. These factors are likely to affect, for example, the pseudomonad populations in a single plant species (Latour, Corberand, Laguerre, Allard, & Lemanceau, 1996). Moreover, it is not yet clarified what could be the impact of PWD in the pine endophytic microbiome. New methodologies such as next‐generation sequencing could be used to assess the endophytic microbiome in pine trees and to analyze its shifts in different PWD symptomatic classes.

## 5 Diversity of Bacteria Associated with *Bursaphelenchus xylophilus*

The first reports relating bacteria and PWN resulted from the observation of bacteria on the surface of the nematode. Since then, different bacterial genera have been isolated from *Bursaphelenchus xylophilus* residing in different pine hosts and also from different countries affected by PWD (Table 3). However, initial reports of the bacterial diversity did not show the presence of bacteria inside *B. xylophilus*, contrary to what has been described for entomopathogenic nematodes (EPN) (Goodrich‐Blair, 2007). More recently, two studies showed the microbiome of PWN by metagenomic techniques (Cheng et al., 2013; Xiang, Wu, & Zhou, 2015).

**Table 3:** Bacterial genera associated with PWN from different countries and different host *Pinus* spp

Country	Bacterial genera carried by PWN	Host pine species	References
Japan	*Bacillus*	*P. densiflora*	Kawazu, Zhang, & Kanzaki (1996)
	*Pseudomonas*		Oku et al. (1980)
China	*Achromobacter*	*P. massoniana*	Wu et al. (2013)
	*Stenotrophomonas*		Wu et al. (2013)
	*Achromobacter*	*P. taiwanensis*	Wu et al. (2013)
	*Ewingella*		Wu et al. (2013)
	*Achromobacter*	*P. thunbergii*	Wu et al. (2013)
	*Buttiauxella*		Zhao et al. (2003)
	*Enterobacter*		Zhao and Lin (2005) and Zhao et al. (2003)
	*Ewingella*		Wu et al. (2013)
	*Leifsonia*		Wu et al. (2013)
	*Pantoea*		Han et al. (2003); Zhao and Lin (2005); Zhao et al. (2003)
	*Peptostreptococcus*		Zhao and Lin (2005); Zhao et al. (2003)
	*Pseudomonas*		Han et al. (2003); Zhao and Lin (2005); Zhao et al. (2003); Zhu, Ye, et al. (2012); Wu et al. (2013)
	*Rhizobium*		Zhu, Ye, et al. (2012)
	*Serratia*		Zhao and Lin (2005); Zhao et al. (2003)
	*Staphylococcus*		Zhao and Lin (2005)
	*Stenotrophomonas*		Tian et al. (2010); Wu et al. (2013)
Korea	*Brevibacterium*	*P. densiflora*	Kwon et al. (2010)
	*Burkholderia*		Kwon et al. (2010)
	*Enterobacter*		Kwon et al. (2010)
	*Ewingella*		Kwon et al. (2010)
	*Serratia*		Kwon et al. (2010)
Portugal	*Burkholderia*	*P. pinaster*	Proença et al. (2010); Vicente et al. (2011)
	*Cronobacter*		Proença et al. (2010)
	*Curtobacterium*		Proença et al. (2010)
	*Enterobacter*		Vicente et al. (2011)
	*Erwinia*		Proença et al. (2010); Vicente et al. (2011)
	*Ewingella*		Proença et al. (2010)
	*Hafnia*		Proença et al. (2010)
	*Janthinobacterium*		Proença et al. (2010)
	*Klebsiella*		Proença et al. (2010)
	*Luteibacter*		Proença et al. (2010)
	*Pantoea*		Proença et al. (2010); Vicente et al. (2011)
	*Pectobacterium*		Vicente et al. (2011)
	*Pseudomonas*		Proença et al. (2010); Vicente et al. (2011)
	*Rahnella*		Proença et al. (2010); Vicente et al. (2011)
	*Serratia*		Proença et al. (2010); Vicente et al. (2011)
	*Yersinia*		Proença et al. (2010)
USA	*Bacillus*	*P. sylvestris* & *P. nigra*	Proença, Fonseca, et al. (2014)
	*Kblebsiella*		Proença, Fonseca, et al. (2014)
	*Pseudoxanthomonas*		Proença, Fonseca, et al. (2014)
	*Serratia*		Proença, Fonseca, et al. (2014)
	*Burkholderia*	*P. sylvestris*	Proença, Fonseca, et al. (2014)
	*Chryseobacterium*		Proença, Fonseca, et al. (2014)
	*Comamonas*		Proença, Fonseca, et al. (2014)
	*Dyella*		Proença, Fonseca, et al. (2014)
	*Enterobacter*		Proença, Fonseca, et al. (2014)
	*Pigmentiphaga*		Proença, Fonseca, et al. (2014)
	*Pseudomonas*		Proença, Fonseca, et al. (2014)
	*Rhizobium*		Proença, Fonseca, et al. (2014)
	*Erwinia*	*P. nigra*	Proença, Fonseca, et al. (2014)
	*Ewingella*		Proença, Fonseca, et al. (2014)
	*Mangroveibacter*		Proença, Fonseca, et al. (2014)
	*Staphylococcus*		Proença, Fonseca, et al. (2014)

The literature sometimes uses, for bacteria isolated in the same way, the terms bacteria associated with and bacteria carried by the nematode, without defining the differences between these concepts. If we take also in consideration that the methodologies used to isolate bacteria from the nematodes differ (directly from the wood or after disinfection, respectively), leading most probably to the isolation of bacteria interacting differently with the nematode, a clear definition of nematode association is not possible.

Oku et al. (1980) found that *Pseudomonas* was carried by PWN, and other studies followed reporting strains from the genus *Bacillus* (Kawazu, Zhang, & Kanzaki, 1996; Kawazu, Zhang, Yamashita, & Kanzaki, 1996) carried by PWN in Japan (Figure 3). PWN isolated from naturally infected *Pinus thunbergii* carried bacteria in average numbers of 290 per adult nematode surface (Guo, Cong, Li, & Zhao, 2002; Guo et al., 2007; Kusunoki, 1987; Zhao, Guo, & Gao, 2000). Moreover, bacteria from the genus *Xanthomonas* were also found to be associated with *B. xylophilus* (Higgins et al., 1999). Researchers in China isolated species from the genera *Pantoea*,* Peptostreptococcus*,* Enterobacter*,* Serratia*,* Staphylococcus*,* Buttiauxella*,* Stenotrophomonas*, and most frequently *Pseudomonas* (Han et al., 2003; Tian et al., 2010; Zhao, Han, Wang, & Han, 2003; Zhao & Lin, 2005). Notably, the genera *Pantoea* and *Pseudomonas* were absent from trees not infected with *B. xylophilus* (Han et al., 2003). Similarly, *Burkholderia arboris*,* Brevibacterium frigoritolerans*,* Enterobacter asburiae*,* Ewingella americana*, and *Serratia marcescens* were found in the Republic of Korea associated with the nematode (Kwon et al., 2010).

Bacteria associated with PWN in Portugal were mainly *Pseudomonas*,* Burkholderia*, and *Enterobacteriaceae* (genera *Yersinia*,* Serratia*,* Ewingella*,* Pantoea*, and *Erwinia*) (Proença et al., 2010). These findings were confirmed by a second study (Vicente, Nascimento, Espada, Mota, & Oliveira, 2011). Bacteria associated with PWN belonging to the genus *Burkholderia* were for the first time reported (Proença et al., 2010). *Burkholderia* spp. are commonly part of endophytic microbial communities from different plants species (Suárez‐Moreno et al., 2012). Another study, also conducted in Portugal, found the genera *Bacillus*,* Citrobacter*,* Enterobacter*,* Escherichia*,* Klebsiella*,* Paenibacillus*,* Pantoea*, and *Terribacillus* associated with PWN (Roriz, Santos, & Vasconcelos, 2011). However, in this last study, the PWN were artificially introduced into pines, and thus, results obtained cannot be directly compared with in situ and ex situ studies (data are not included in Table 3). Most of the studies mentioned were based on classical microbiological methods for studying the nematode‐associated bacteria. More recently, the bacterial diversity of *B. xylophilus* and *B. mucronatus* (nonvirulent species of the genus *Bursaphelenchus*) was investigated using next‐generation sequencing and showed that the most abundant genera were *Stenotrophomonas* and unclassified genera belonging to the families *Pseudomonadaceae* and *Rhizobiaceae* (Xiang et al., 2015). These findings partly confirm previous studies in which, by cultivation methods, the genus *Stenotrophomonas* was identified as part of community from *B. xylophilus* (Wu et al., 2013).

Proença, Fonseca, et al. (2014) and Proença, Nobre, et al. (2014) investigated the diversity of bacteria carried by PWN isolated in several pine species of the United States of America, the native home of the nematode (Proença, Fonseca, et al., 2014; Proença, Nobre, et al., 2014). Similarly to previous reports, the majority of bacteria carried by PWN in the USA belonged to the family *Gammaproteobacteria* (79.9%); additionally, the genera *Chryseobacterium* and *Pigmentiphaga* were found for the first time (Table 3) (Proença, Fonseca, et al., 2014; Proença, Nobre, et al., 2014). The overall diversity of bacteria was found to depend on different national forest areas, country or the *Pinus* spp. sampled. Nonetheless, strains belonging to the genus *Pseudomonas* were identified as associates with PWN from all *Pinus* spp. sampled from all the countries (Proença, Fonseca, et al., 2014; Proença, Nobre, et al., 2014). On the other hand, the genus *Pseudomonas* includes strains that can be isolated in many different environments and it is difficult to support a specific association with the nematode.

At this point, one central question is the origin of the bacteria carried by the nematode. Two hypotheses can be considered: they are pathogenic bacteria promoting PWD, carried by PWN, invading the host pine trees, and cannot enter other ways; alternatively, these bacteria were already in the tree (as endophytic bacteria or carried by other organisms) and, the presence of the PWN activated a mutualistic effect. On the other hand, bacteria carried by the nematode might be endophytes that adhere to the nematode cuticle (as a support or as food) and are not participating in PWD.

## 6 Nematode–Bacteria Interactions

When elucidating relationships between PWN and its bacteria, it might be noteworthy to analyze the relationship between bacteria and other nematodes. Nematode–bacteria interactions can be beneficial—mutualistic relationship, or harmful—parasitic/pathogenic relationship, or neutral. As an example, *Caenorhabditis elegans*, a bacteria‐feeding nematode, is a model for human infectious diseases and allows evaluation of pathogenicity mediated by bacteria (Couillault & Ewbank, 2002; Irazoqui, Urbach, & Ausubel, 2010; Pukkila‐Worley & Ausubel, 2012; Waterfield et al., 2008). *Bacillus nematocida* B16 is harmful to *C. elegans* by production of volatile organic compounds (VOC) attracting the worm and once inside the nematode‐intestine, the bacterium secretes two proteases that lead to nematode death (Niu et al., 2010). Mutualistic relationships have been studied in EPN of the genera *Heterorhabditis* or *Steinernema* and *Gammaproteobacteria* endosymbionts of the genera *Photorhabdus* and *Xenorhabdus*, respectively (Forst & Nealson, 1996; Goodrich‐Blair & Clarke, 2007; Murfin et al., 2012; Ryss, Kulinich, Turitsin, & Mazurin, 2011). EPN are able to use bacterial symbionts as a food source or to use essential nutrients produced by bacteria that are critical for efficient nematode proliferation (Hu & Webster, 2000; Isaacson & Webster, 2002; Murfin et al., 2012; Ryss et al., 2011). For example, bacteria have been shown to be involved in killing insects, destroying insect body structures, and releasing nutrients that in turn are important for EPN development and proliferation (Goodrich‐Blair & Clarke, 2007).

Plant cell‐wall‐degrading enzymes have been demonstrated to be important for host–parasite interactions. *Bursaphelenchus xylophilus* produces cellulases (β‐1,4‐endoglucanses) and pectate lyases that permit invasion of plant tissues (Kikuchi, Jones, Aikawa, Kosaka, & Ogura, 2004; Kikuchi, Shibuya, Aikawa, & Jones, 2006; Kikuchi et al., 2011). Because such proteins are of bacterial and fungal origin, this suggests horizontal gene transfer (HGT) from bacteria and fungi to nematodes (Kikuchi et al., 2011; Scholl, Thorne, McCarter, & Bird, 2003). Such unexpected coevolutionary processes stimulated the interest in the study of plant parasitic nematodes (PPN) and their interactions with bacteria (Bird & Koltai, 2000; Scholl & Bird, 2011).

## 7 Roles of Bacteria Carried by PWN in PWD

The observation of bacteria associated with nematodes led to the question what could be the function of bacteria carried by the nematode in PWD. Several studies showed a relationship between PWD and the number of bacteria in the tree tissues, some related to the diversity of the bacteria, and some showed that axenic PWN were not pathogenic. None of the effects of bacteria proved to be universal and not a single study identified bacteria as the only agent responsible for PWD. Therefore, it is relevant to discuss what might be the roles of these bacteria. These roles may be related to the origins of these bacteria, which are not completely defined, as we discussed above. When nematode‐associated bacteria are described from nematodes that were isolated from infected trees, it is plausible that these bacteria may be endophytes, but it is also possible that these may have intimate interaction with the nematodes, and have a mutualistic activity in PWD.

In the following two sections, we will discuss what could sustain a synergetic effect with PWN and what could bestow bacteria with functions of tentatively controlling nematodes inside the tree.

### 7.1 Synergistic effect of bacteria with PWN on PWD development

Several studies have supported the idea of bacterial roles in PWD, acting as phytopathogens for *Pinus* spp. (Kawazu & Kaneko, 1997; Kawazu, Zhang, & Kanzaki, 1996; Kawazu, Zhang, Yamashita, et al., 1996; Oku et al., 1980). Oku et al. (1980) observed rapid wilting of pine trees and suggested that this was a consequence not only of the nematode but also of the bacteria transmitted by the PWN. The authors found that these bacteria belonged to the genus *Pseudomonas* and were involved in production of toxins (Oku et al., 1980). Unfortunately, the ability of bacteria to produce toxins was lost 2 months after isolation indicative for extrachromosomal provenience of the respective toxin genes (Oku et al., 1980).

Phenylacetic acid (PA)‐producing bacteria were suggested as pathogens and might be involved in the disease mechanism of PWD (Kawazu, Zhang, & Kanzaki, 1996; Kawazu, Zhang, Yamashita, et al., 1996). In Japan, *Bacillus cereus* strain HY‐3 carried by pinewood nematode was shown to produce PA (Kawazu, Zhang, & Kanzaki, 1996; Kawazu, Zhang, Yamashita, et al., 1996). PA permits generation and accumulation of benzoic acid (BA) and its conjugates in suspension of cultured cells of *Pinus thunbergii* as well as in 3 years old saplings (Kawazu, Zhang, & Kanzaki, 1996; Kawazu, Zhang, Yamashita, et al., 1996).

Based on these works, several studies were performed focusing on the pathogenic role of bacteria carried by the nematodes through the use of aseptic nematodes. According to Han et al. (2003), the inoculation of aseptic nematodes (*B. xylophilus* or *B. mucronatus*) did not cause browning or wilt of *P. thunbergii* calli or seedlings (Han et al., 2003). However, the combination of axenic nematodes with *Pseudomonas* strains (Njh and Njt) caused severe symptoms. Notably, browning of the plants was also observed when filtered culture liquid of *Pseudomonas* strains Njh and Njt was applied to the calli. These authors suggested that co‐infection of PWN and bacteria (or toxins produced by bacteria) play an active role in the development of PWD (Han et al., 2003). In a different study, axenic *B. mucronatus* (a nonpathogenic species) and axenic *B. xylophilus* did not cause wilt, but when combined with pathogenic bacteria, both species of *Bursaphelenchus* were able to induce PWD (Zhao et al., 2009). Furthermore, Zhao, Tao, Ju, Wang, and Ye (2011) showed that PWD was caused by a combination of axenic PWN with each of seven bacterial isolates of the genera *Bacillus* and *Stenotrophomonas* (Zhao et al., 2011). In contrast, inoculations of aseptic microcuttings or seedlings of *Pinus densiflora* with axenic PWN inoculated with *Rhizobium* sp. and *Pseudomonas* sp. did not cause wilt (Zhu, Ye, et al., 2012).

Remarkably, Zhao et al. (2003) did not find any bacteria in healthy pine trees, but found bacteria carried by PWN from diseased trees. In that study, 24 bacterial strains were isolated and 17 of them were identified as phytotoxin producers. The majority of these phytotoxin‐producing bacteria belonged to the genus *Pseudomonas* (Zhao et al., 2003). These authors also performed inoculations of black pine seedlings and pine trees in greenhouse and field inoculations and showed that the presence of both PWN and its associated phytotoxin‐producing bacteria were necessary for causing the disease (Zhao et al., 2003). The number of PWN and bacteria were found to increase when needles of black pine trees changed into yellow or brown and when the studied trees were completely wilted (Xie, Ju, & Zhao, 2004; Xie & Zhao, 2008).

Other authors proposed a mutualistic symbiotic relationship between the PWN and its associated bacteria of the genus *Pseudomonas* (Zhao & Lin, 2005). For instance study, PWN reproduction was promoted by 10 strains of the genus *Pseudomonas* (previously isolated as bacteria carried by PWN (Zhao et al., 2003), namely *P. fluorescens* (GcM5‐1A, ZpB2‐1A), *P. putida* (GcM6‐2A, GcM6‐1A, ZpB1‐2A, GcM2‐3A), *Pseudomonas* sp. (HeM‐139A, HeM2A, HeM1A), and *P. cepacia*, reclassified as *Burkholderia cepacia* (GcM1‐3A), but 19 strains showed an inhibitory effect on PWN reproduction. On the other hand, the presence of PWN promoted the multiplication of 18 of the 29 bacterial strains.

The strain *P. fluorescens* GcM5‐1A (Zhao et al., 2003) was evaluated in terms of toxicity to suspension cells and seedlings of *Pinus thunbergii* (Guo et al., 2007). According to Guo et al. (2007), two cyclic dipeptides, cyclo(‐Pro‐Val‐) and cyclo(‐Pro‐Tyr‐), were secreted by strain GcM5‐1A and showed toxic activity to both suspension cells and seedlings. Moreover, a 50 kDa protein was secreted by the same strain, purified from the culture, and N‐terminal sequencing revealed the peptide to be similar to flagellin (Guo et al., 2008). Flagellin as well as the two cyclic dipeptides, showed toxicity to suspension cells and seedlings of *P. thunbergii* (Guo et al., 2008). Later, Zhang et al. (2012) showed that flagellin when added to calli of *P. thunbergii* in vitro was able to promote PWN proliferation and that of its associated bacteria. Remarkably, inoculations on dead calli of *P. thunbergii,* pretreated with flagellin, yielded much higher population numbers of PWN and *Pseudomonas fluorescens* GcM5‐1A than on living calli (Zhang et al., 2012). Moreover, a recombinant protein of the Dyp‐type peroxidase family, with or without the putative signal peptide of *P. fluorescens* strain GcM5‐1A, showed toxicity to both suspension cells and seedlings of *P. thunbergii* (Kong et al., 2010).

*Burkholderia arboris* KRICT1 carried by PWN, produced the siderophore pyochelin which is phytotoxic to the seedlings and calli of *P. densiflora* (Le Dang et al., 2011). Pyochelin was shown to have stronger phytotoxicity compared to PA produced by *Bacillus* spp. and might be involved in the PWD wilting process (Le Dang et al., 2011). Thus, these results supported the hypothesis of the role of bacteria and their toxins in the development of PWD.

As pointed out by Jones et al. (2008), some of the above studies have limitations because the tests were mostly performed in vitro*,* and used calli, young trees, or seedlings. Additionally, only a few strains of the microbial diversity carried by PWN were evaluated (Jones et al., 2008). Wu et al. (2013) showed that some bacterial species and their carbon metabolism were related to virulence of *B. xylophilus*, that is, bacteria with a higher carbon utilization correlated with highly virulent *B. xylophilus* (Wu et al., 2013). Recently, strains from the genera *Serratia*,* Enterobacter*, and *Pantoea* associated with PWN showed to be able to induce PWD symptoms in *P. pinaster* seedlings (Vicente et al., 2012).

Although mutualistic bacteria were suggested to be necessary to promote PWD, the way these bacteria enter in the tree was not clarified, as mentioned above. On the other hand, it was also suggested that bacteria are able to produce toxic compounds involved in nematotoxicity against PWN (Paiva et al., 2013; Proença, Espírito Santo, Grass, & Morais, 2012a,b). The role of bacteria carried by PWN that plays on PWD may be double‐edged as outlined next.

### 7.2 Protective role of bacteria for pine trees

The majority, if not all the species mentioned to be related with the PWN pathogenicity, are regular components of endophytic communities and are common in soil and water environments. It is also known that some studies recognized several secondary metabolites produced by endophytic bacteria to be involved in a broad spectrum of roles, for example, having biotechnological potential, producing antibiotics, acting as biological control agents against plant pathogens as well as promoting plant growth (Figure 3) (Qin, Xing, Jiang, Xu, & Li, 2011).

In 2010, the bacterial diversity associated with PWN from *Pinus pinaster* was reported in Portugal (Proença et al., 2010). As described in section 6, isolates were identified and all belonged to the *Beta* or *Gammaproteobacteria*, except for one gram‐positive *Actinobacteria* strain. These bacteria were suggested to play a role in physiological plant responses, that could be used against invasion, because 60–100% of the isolates, depending on sampled area, readily produced siderophores and at least 40% produced lipases (Proença et al., 2010). Siderophores, as iron chelators, may have a dual effect: either they potentially promote plant growth and plant protection, or they are typical virulence factors since they were suggested to inhibit iron uptake by plants (Gamalero & Glick, 2011).

The ability of bacteria to produce lipases can be related to a beneficial role of bacteria protecting trees from PWD because plant lipids as well as their hydrolysis products have been described as playing part in plant defense (e.g., monolaurate and oleic acids) (Hunzicker, 2009; Kwon et al., 2009; Raffaele, Leger, & Roby, 2009). According to Proença et al. (2010) from a group of isolates carried by PWN, a few bacterial strains were able to produce proteases (Proença et al., 2010) and were explored later for nematicidal properties (Paiva et al., 2013).

More recently, two bacterial genomes of nematotoxic isolates, *Pseudomonas* sp. M47T1 and *Serratia* sp. M24T3, were sequenced (Proença et al., 2012a,b). Both draft genomes showed multiple genes potentially involved in virulence and nematotoxic activity, such as the genes coding for colicin V and bacteriocin biosynthesis (Proença et al., 2012a,b). On the other hand, the genus *Serratia* was already reported as pathogenic to the pine sawyer beetle *Monochamus* (Shimazu, 2009). Recently, nematotoxicity assays of various strains carried by PWN from Portugal were performed. In contrast, the most active strain, *Serratia* sp. A88copa13, was selected for further studies (Paiva et al., 2013). Some chemical compounds and other bio‐products produced by bacteria carried by nematode have been suggested to control PWD as detailed in the next section.

## 8 Microbial Products Active Against PWNs

In order to find a strategy for control of PWD, several studies developed bioassays against the nematode *Bursaphelenchus xylophilus* using proteases, essential plant oils, or chemical products (Figure 3). Proteases represent a class of enzymes, found in plants, animals, and microorganisms, and may be divided into two groups: (i) intracellular proteases, with physiological roles inside the organisms, for example, altering protein turnover, sporulation and conidial discharge, germination, enzyme modifications, nutrition, regulation of gene expression; (ii) extracellular proteases, modifying protein catabolism with roles outside the microorganisms, and also acting as exotoxins causing pathophysiological processes (Rao, Tanksale, Ghatge, & Deshpande, 1998). Such hydrolytic secreted enzymes produced by some gram‐positive bacteria are indeed involved in the degradation of nematode components (Cox, Kusch, & Edgar, 1981; Decraemer, Karanastasi, Brown, & Backeljau, 2003; Huang et al., 2005). Alkaline serine proteases have been reported to be produced by *Brevibacillus laterosporus* strain G4 (30 kDa designated BLG4) and from *Bacillus nematocida* (28 kDa) (Huang et al., 2005; Qiuhong et al., 2006). Nevertheless, both proteases were not the only virulence factors responsible for the observed nematicidal activities from these bacteria. According to Lian et al. (2007), an extracellular cuticle‐degrading protease from *Bacillus* sp. strain RH219 designated Apr219 was reported to be an important nematicidal factor, killing nematodes within 12 h (Lian et al., 2007).

The gram‐negative bacterium *Stenotrophomonas maltophilia* G2, isolated from soil, has been suggested to possess nematotoxic activity against *B. xylophilus*, killing 65% of the nematodes within 24 h of incubation (Huang et al., 2009). Analysis of possible virulence factors revealed the presence of an extracellular 28 kDa serine protease able to digest the nematode cuticle (Huang et al., 2009). Notably, among the hydrolytic enzymes, serine proteases have recently been shown to be very important in the penetration and digestion of nematodes by nematode‐trapping fungi (Ahman et al., 2002; Lopez‐Llorca, Olivares‐Bernabeu, Salinas, Jansson, & Kolattukudy, 2002; Wang, Yang, & Zhang, 2006; Yang et al., 2005).

The production of serralysin‐like proteases was detected in several *Serratia* strains related to insects (Kim, Golyshin, & Timmis, 2007). A metalloproteinase is also produced by the entomopathogenic bacteria from the genera *Xenorhabdus* and *Photorhabdus* (*P. luminescens*), both symbiotic with their respective nematodes (Massaoud, Marokházi, Fodor, & Venekei, 2010).

Serralysin and serine protease were found to be extracellular proteases of *Serratia* sp. A88copa13 (Paiva et al., 2013). In comparison with previously described proteases, this extracellular 70 kDa serine protease which is mostly responsible for nematoxicity associated with culture supernatant, is phylogenetically and biochemically different from the ones that were characterized before (Paiva et al., 2013). Moreover, nematicidal activities of the proteases differed according to species of the genus *Bursaphelenchus*, supporting the utility of this protease in a primary screen for nematode differentiation based on nematotoxicity (Paiva et al., 2013). This study pointed out that the bacteria carried by PWN possibly promote an efficient attack against PWN since they are able to kill the pathogen, and thus might play a role in the defense of pine trees against *B. xylophilus* (Paiva et al., 2013).

## 9 Bacterial Genomics Relates to PWN

Next‐generation sequencing (NGS) may overcome challenges related to species classification, and physiological and biochemical characterization of bacteria as well as elucidating interaction mechanisms between organisms. Furthermore, genome analysis potentially might lead to new discoveries resulting in biotechnological applications (Chun & Rainey, 2014; Kämpfer & Glaeser, 2012). At time of writing, 20 endophytic bacterial genomes were completely sequenced belonging to the classes of *Actinobacteria*,* Alphaproteobacteria*,* Bacilli*,* Betaproteobacteria*, and *Gammaproteobacteria*, isolated from different environments as summarized in Table 4.

**Table 4:** Endophytic bacterial strains from different hosts with completely sequenced genomes

Endophytic class	Endophytic strain	Isolation source	References
*Actinobacteria*	*Microbacterium testaceum* StLB037	Potato leaves	Morohoshi, Wang, Someya, and Ikeda (2011)
*Alphaproteobacteria*	*Gluconacetobacter diazotrophicus* Pal5	Sugarcane root	Bertalan et al. (2009)
	*Azospirillum* sp. B510	Rice stem	Kaneko et al. (2010)
*Bacilli*	*Bacillus subtilis* BSn5	*Amorphophallus konjac* calli tissue	Deng et al. (2011)
*Betaproteobacteria*	*Azoarcus* sp. BH72	Kallar grass roots	Krause et al. (2006)
	*Burkholderia phytofirmans* PsJN	Onion root	Weilharter et al. (2011)
	*Burkholderia* sp. KJ006	Rice root	Kwak et al. (2012)
	*Variovorax paradoxus* S110	Potato plant	Han et al. (2011)
*Gammaproteobacteria*	*Enterobacter cloacae* subsp. *cloacae* ENHKU01	Pepper	Liu et al. (2012)
	*Enterobacter* sp. 638	Poplar stem	Taghavi et al. (2009, 2010)
	*Enterobacter* sp. R4‐368	Roots of *Jatrophacurcas* L.	Madhaiyan, Peng, and Ji (2013)
	*Klebsiella pneumoniae* 342	Maize stem	Fouts et al. (2008)
	*K. variicola strain DX120E*	Sugarcane root	Lin et al. (2015)
	*Serratia proteamaculans* 568	Poplar root	Taghavi et al. (2009)
	*S. plymuthica* AS9	Rapeseed roots	Neupane, Finlay, Alström, et al. (2012)
	*S. plymuthica* AS12	Rapeseed roots	Neupane, Finlay, Kyrpides, et al. (2012)
	*S. plymuthica* AS13	Rapeseed roots	Neupane, Högberg, Alström, et al. (2012)
	*Stenotrophomonas maltophilia* R551‐3	Poplar stem, root, and rhizosphere	Taghavi et al. (2009)
	*Pseudomonas putida* W619	Poplar stem and root	Taghavi et al. (2009)
	*Pseudomonas poae* RE*1‐1‐14	Endorhiza of the sugar beet	Müller et al. (2013)

It is important to highlight that many works have looked at the bacteria leaving in/on plants as symbionts of the tree. The existing endophytic genomes were chosen in order to address possible beneficial plant growth‐promoting roles by identification of required genes and also to discover possible new strategies for agriculture biotechnology. Moreover, the strains reported have been demonstrated, for example, to have protective roles against fungal and bacterial phytopathogens (*Xanthomonas oryzae* and *X. albilineans*) (Bertalan et al., 2009; Kaneko et al., 2010). Comparison of bacterial endophytic genomes with those from different environments (e.g., clinical, soil, etc.) will likely allow a better understanding of requisites for successful colonization and other symbiotic interactions. As an example, *Stenotrophomonas maltophilia* which is a known endophyte found in/on plants with a worldwide distribution is used. While being an endophyte, a large number of epiphytic fitness genes such as those associated with pili, fimbriae, and adhesions were found in the genome (strain RR‐10) (Zhu, Liu, et al., 2012). On the other hand, *S. maltophilia* that have been isolated from clinical and from soil samples, proved to be nosocomial multidrug‐resistant microorganisms, recognized to be involved in pathogenesis in humans. The complete genome of strain *S. maltophilia* K279a was sequenced and several genes involved in drug resistance, adhesion, biofilm formation, and mobile elements were identified, suggesting possible roles in pathogenicity (Crossman et al., 2008). Moreover, in several nosocomial strains of this species, proteases in general and extracellular proteases specifically, have been characterized and implicated in disease (Travassos et al., 2004). The major extracellular protease (Peptidase S8 [Smlt0861]) of strain K279a was suggested to be a virulence determinant (Windhorst et al., 2002). Notably, a different strain, *S. maltophilia* G2 isolated from soil, was also able to kill nematodes by producing extracellular serine protease (Huang et al., 2009). Future genomic analysis in combination with physiological and biochemical studies will likely allow for a deeper understanding of molecular modes‐of‐action related to various pathogenic processes. Possibly, the initial comparison between genomes (draft or completed) will elucidate genetic differences between genomes reflecting the diversity of assumed roles by strains of the same bacterial species from different environments. In case of *Serratia marcescens*, a second example, this reaches from being a plant growth promoter (Dong, Gu, Guo, Xun, & Liu, 2014), specially under plant stress, to acting as a phytopathogen through the acquisition of genetic mobile elements with an integron harboring genes that take part in the histidine metabolism (Ovcharenko et al., 2010) or responding to environmental stimuli (Zhang et al., 2005).

Cheng et al. (2013) using metagenomic analysis of the PWN microbiome showed a symbiotic relationship between PWN and its associated bacteria for xenobiotics degradation (Cheng et al., 2013). Comparing the metagenomes obtained from the nematode kept in culture, the nematode from a symptomatic tree, and the genome from Portuguese *Serratia* sp. M24T3 (Proença et al., 2010, 2012a,b), Cheng and collaborators found abundant genes coding for enzymes involved in metabolism of xenobiotics degradation. In the metagenomes, the presence of a complete pathway of α‐pinene degradation, which was identified in the genome of *Serratia* sp. M24T3, was also found. This pathway, additional to the cytochrome P450 detoxification system of the nematode, could sustain nematode survival in the presence of α‐pinene, β‐pinene, and the secondary metabolic compounds which are relatively abundant in pine trees.

Advances in molecular biology and the introduction of new methodologies will likely provide a better understanding of all the factors involved in pathogenicity of pathways of nematotoxin production as well as of the complex bacterial–nematode–plant interactions (Figure 3). Finally, based on bacterial genome sequencing and the application of this knowledge, the discovery of new products, for example, microbial toxins or enzymes with new properties, can be anticipated to promote and formulate commercial nematicidal agents (Tian, Yang, & Zhang, 2007).

## 10 Concluding Remarks

Bacteria are now thought to play a role in the development of pine wilt disease (PWD), for bacteria belonging to several different genera have been found associated with the pinewood nematode (PWN). Molecular methodologies and the new era of high‐throughput genomics have opened a fresh perspective to the challenge, by recognizing the interactions between different organisms involved in the disease (Box 2).

Box 2 **Future Focus Questions**

1

- Which nematode‐associated bacteria promote PWD? Conversely, which (nematode‐associated) bacteria suppress PWD?
- Where do the nematode‐associated bacteria originally come from? Are bacterial species simply “carried” by the PWN or indeed “associated,” meaning involved in modulating the worm's potential for virulence? Are the bacteria involved specific species capable of engaging in this relationship? If yes, how are these strains selected locally?
- What is the functional core microbiome associated with the nematode‐bacteria association? What factors provided by the bacteria (and nematode) support this inter‐kingdom relationship?
- How can such bacteria best be harnessed for biocontrolling the PWN?

Forests constitute major microbial ecosystems, with trees being chief inhabitants of many terrestrial ecosystems, providing various dedicated ecological niches. The association between bacteria and plants and the mechanisms leading to the selection of endophytic bacteria have now been acknowledged. The functions of these microorganisms range from plant protection to growth promotion. Conversely, there is also a wide variety of phytopathogenic bacteria responsible for various diseases, yet, there is little information on those that might affect forest trees, in the context of the trees' diverse endophytic communities.

The presence of prokaryotic genes in the genome of different nematode families suggests a close relationship between nematodes and bacteria in the evolutionary past. The best documented cases of symbiotic relationships are those of the entomopathogenic nematodes of the families Heterorhabditidae and Steinernematidae with endosymbiont bacteria of the genera *Xenorhabdus* and *Photorhabdus*, respectively. Nematodes of the genus *Bursaphelenchus* appear to have acquired genes of fungal cellulases and bacterial glucanases. In *Bursaphelenchus xylophilus*, the mutualistic relationship is still unclear. The words carried and associated are used in many studies without a concrete definition. Understanding whether, a bacterial species is simply “carried” by the PWD nematode or indeed “associated,” meaning that is involved in modulating the worm's potential for virulence in a specific manner, is a major question. Up to now, the diversity of the bacteria associated with PWN, obtained from infected trees, has been studied in different countries. In Japan, the species are predominantly of the genus *Bacillus*, in China of the genera *Burkholderia*,* Enterobacter*,* Pantoea*,* Peptostreptococcus*, and *Pseudomonas*, and in Korea of the genera *Brevibacterium*,* Burkholderia*,* Enterobacter*,* Ewingella*,* Serratia*,* Bacillus*, and *Pseudomonas*. Bacteria of the genus *Xanthomonas* are also referred to as associated with *B. xylophilus*. In Portugal, the bacterial community seems to differ according to the forest area, and only strains belonging to the families *Enterobacteriaceae* and *Pseudomonadaceae* were commonly found in all the sampled areas. The strains of the family *Enterobacteriaceae* belong to different species, the most abundant being *Cronobacter dublinensis*,* Ewingella americana*,* Pantoea cypripedii*,* Serratia marcescens*,* S. plymuthica*, and species of the genus *Erwinia*. Most bacteria of the family *Pseudomonadaceae* belong to the species *Pseudomonas koreensis*,* P. lutea*,* P. moorei*, and *P. putida*. Some strains of the *Burkholdariaceae* family, especially of the species *Burkholderia tuberum*, were also found. Where these nematode‐associated bacteria originally come from, and how they become associated with the nematode, still needs an answer. The bacterial species found carried by the PWN are also present in the pine trees endophytic community and in the environment. This makes us to question if bacteria engaging in relationship with the nematode, and potentially involved in PWD, could be specific strains or could be various strains of a single species.

The function of these bacteria regarding maritime pine (*P. pinaster*) is not yet defined. It has been proposed that one or more bacteria associated with the PWN may be involved in nematode virulence, although the mechanism of pathogenicity is not yet fully understood. One needs to know what are the factors provided by bacteria and those provided by the nematode that signify and support the inter‐kingdom relationship. The production of phytotoxins by strains associated with PWN in seedling assays revealed that the presence of bacteria increases nematode pathogenicity. However, there are also bacteria which have nematicidal potential and are able to eliminate nematodes in vitro. In this process, the enzymes involved are proteases and lipases, and eventually siderophores and surfactants excreted by bacteria. The most relevant nematicidal products are proteases (serine and metalloproteases). The serine proteases, produced by different species such as *Bacillus nematocida*,* Brevibacillus laterosporus*, and *Serratia marcescens*, were identified as virulence factors of *Bursaphelenchus xylophilus*, while metalloproteases are produced by bacteria mutualistic with entomopathogenic nematodes and are responsible for the death of insect larvae.

The partial genome sequences of species from the bacterial families *Enterobacteriaceae* and *Pseudomonadaceae*, both *Gammaproteobacteria*, revealed not only the presence of genes potentially involved in nematicidal activity but also a set of genes associated with a symbiotic adaptation to plants and to the promotion of their growth. The two studied strains (*Serratia* sp. M24T3 and *Pseudomonas* sp. M47T1) possess genes coding for nitrogen regulatory protein P‐II (ammonia assimilation) and acetoin‐diacetyl reductase, which might aid in promoting plant growth and providing protection against fungal and bacterial infections. These two strains encode genes for the biosynthesis of bacteriocins (antimicrobial peptides that destroy or inhibit the growth of other bacteria) and for colicin V, a high–molecular‐weight bacteriocin that could be involved in pathogenicity against to *B. xylophilus*.

Thus far, there is no unequivocal consensus on the role of bacteria associated with PWN. Contradictory findings were reported on the strict presence of microorganism in every case of PWD. The evidence for a true association between bacteria and *B. xylophilus*, and whether this would have any consequence for the development of the disease, is not clear. There are many potential pathogens, but not a single unequivocal specific one. The diversity of bacteria associated with the nematode proved to be different in different sampling sites. The comprehensive analysis of both proteomes and genomes of bacteria and PWN are necessary. Some PWN isolates promoted faster plant decay in the presence of some bacterial species, particularly of the genus *Pseudomonas*. On the other hand, some bacterial strains have the capacity to cause the death of *B. xylophilus*. Furthermore, *Serratia* and *Pseudomonas* strains feature in their genome potential plant growth‐promoting genes. The focus on these interactions is relatively recent and will require more experimental information in order to clarify the role of bacteria in PWD. All in all, we need to clarify whether there are bacteria involved in modulating the worm's potential for virulence, and if this relationship is established by one or more specific bacterial species, as part of a functional core microbiome. If so, past studies did not elucidate the origin of these bacteria. Knowing the factors provided by bacteria (and nematode) that support this inter‐kingdom relationship will possibly lead to the selection of the bacteria best harnessed for biocontrolling the nematode in areas affected by PWD.

## Conflict of Interest

The authors declare no conflict of interests for this article.
